# Supplementary material for: Recognition of BRAF by CDC37 and Re-Evaluation of the Activation Mechanism for the Class 2 BRAF-L597R Mutant
Source: Biomolecules. 2022 Jun 28;12(7):905. doi: 10.3390/biom12070905 (PMC9313131; doi:10.3390/biom12070905)
Supplement: Supplementary file 1 [file biomolecules-12-00905-s001.zip › biomolecules-1782743-supplementary.pdf]

Supplementary Figure S1

Cdc37 construct	Cdc37 Structure	Binding partner	Binding parameters	Refer to Fig
Full-length	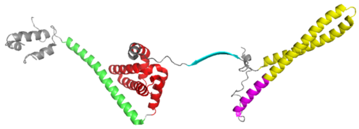	sBraf	*Kd* = 1.0 μM	1A
Full-length	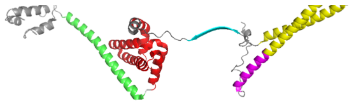	sBrafV600E	*Kd* = 0.41 μM.	1B
Full-length	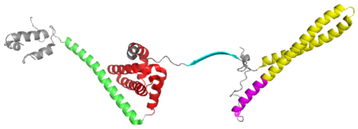	sBrafV600E+ AMPPNP	NB	1C
Full-length	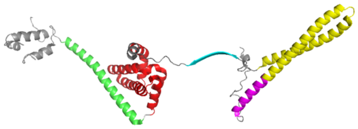	sBrafV600E+ ADP	NB	1D
N 1-120	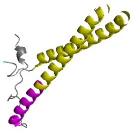	sBrafV600E	*Kd* = 278 μM	2B
C-terminal Domain 273-348	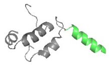	sBrafV600E	*Kd* = 104 μM.	2C
Middle domain 148-269	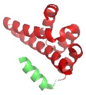	sBrafV600E	NB	2D
Cdc37 TrpZip	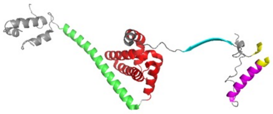	sBrafV600E	*Kd* = 0.5 μM.	3A
Cdc37 Δ(CC-βS)	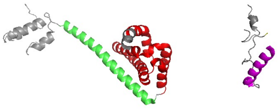	sBrafV600E	*Kd* = 0.61 μM	3B

Supplementary Figure S1 continued

Cdc37 construct	Cdc37 Structure	Binding partner	Binding parameters	Refer to Fig
Full-length	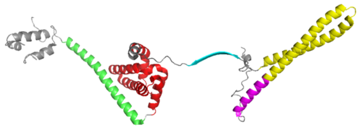	sBrafV600E	*K*d = 0.41 μM.	1B
Cdc37 Δ(7-CC-βS-272-285)	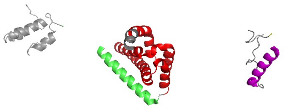	sBrafV600E	*K*d = 1.03 μM.	3C
Cdc37 N^mC	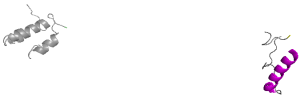	sBrafV600E	*K*d = 145 μM.	3D
Cdc37 N^m(+14)-C	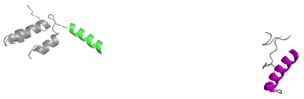	sBrafV600E	*K*d = 8.7 μM	4A
Cdc37 N^m(+6)-C	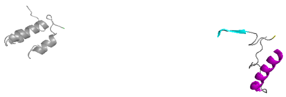	sBrafV600E	*K*d = 18 μM	4B
Cdc37 N^m(+41)-C	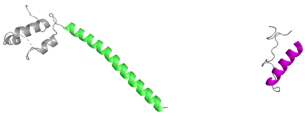	sBrafV600E	*K*d = 1.99 μM	4C
Cdc37 1-343	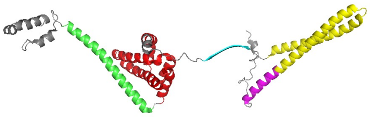	sBrafV600E	*K*d = 0.76 μM	5A
Cdc37 V343A	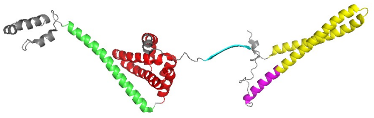	sBrafV600E	*K*d = 0.9 μM.	5B
Cdc37 V343R	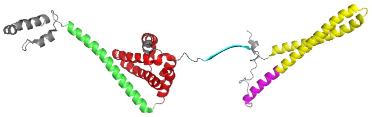	sBrafV600E	*K*d = 1.3 μM.	5C

Supplementary Figure S1 continued

Cdc37 construct	Cdc37 Structure	Binding partner	Binding parameters	Refer to Fig
Full-length	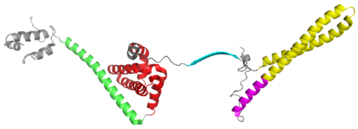	sBrafV600E	*K*d = 0.41 μM	1B
Cdc37 1-342	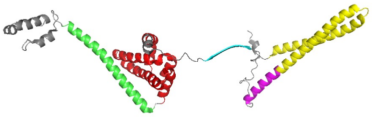	sBrafV600E	*K*d = 45.7 μM	5D
Cdc37 W342A	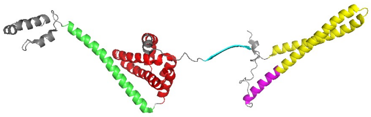	sBrafV600E	*K*d = 94.3 μM	5E
Cdc37 W342R	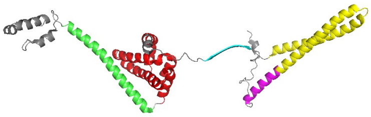	sBrafV600E	NB	5F
Full-length S13E	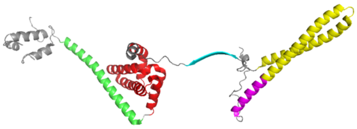	sBrafV600E	*K*d = 0.30 μM	6A
Cdc37 N22A	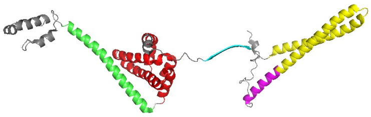	sBrafV600E	*K*d = 27.3 μM	7A
Cdc37 N22R	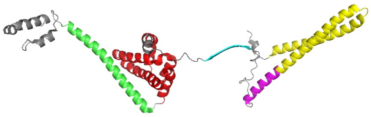	sBrafV600E	*K*d = 14.2 μM	7B

Supplementary Figure S1 continued

Cdc37 construct	Cdc37 Structure	Binding partner	Binding parameters	Refer to Fig
Full-length	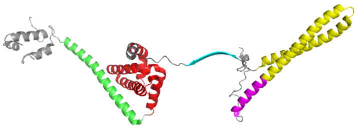	sBrafV600E	*K*d = 0.41 μM	1B
Full-length L28R	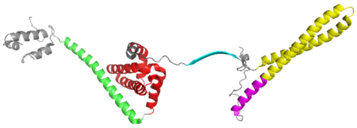	sBrafV600E	NB	8B
Full-length L28A	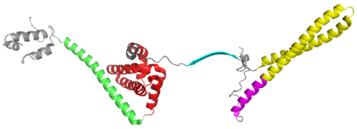	sBrafV600E	*K*d = 16.9 μM.	8C
Full-length W31K	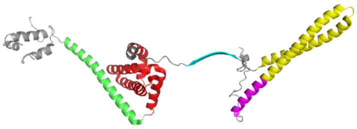	sBrafV600E	NB	8D
Full-length W31A	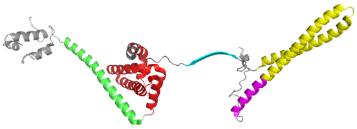	sBrafV600E	*K*d = 2.1 μM	8E
Full-length S27K	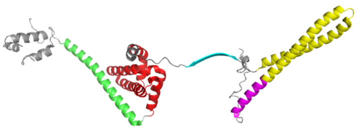	sBrafV600E	NB	8F
Full-length S27A	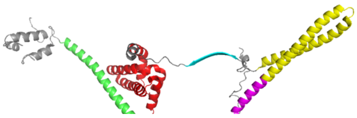	sBrafV600E	*K*d = 1.6 μM.	8G
Full-length Q47R	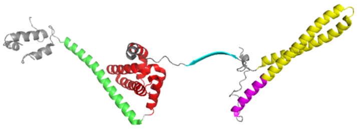	sBrafV600E	*K*d = 0.3 μM	8H
Full-length Q347A	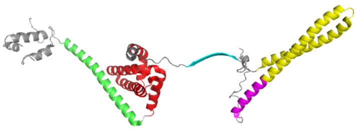	sBrafV600E	*K*d = 0.35 μM.	8I

Supplementary Figure S1 continued

Cdc37 construct	Cdc37 Structure	Binding partner	Binding parameters	Refer to Fig
Full-length	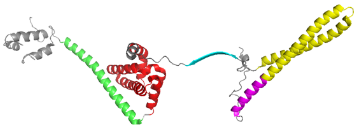	sBrafV600E	*K*d = 0.41 μM	1B
Full-length	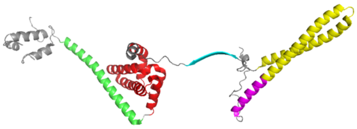	sBrafV600E T599E-V600E-S602D	*K*d = 0.47 μM	9A
Full-length	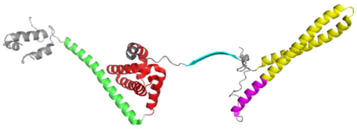	sBrafV600E T599W-V600E	*K*d = 0.23 μM	9B
Full-length	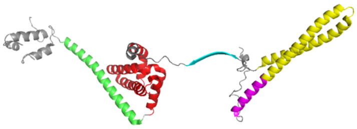	sBraf F595A	*K*d = 0.5 μM	10A
Full-length	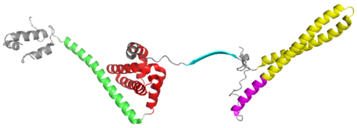	sBraf F595R	*K*d = 1.4 μM	10B
Full-length	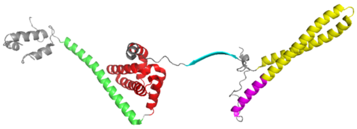	sBraf D594V	*K*d = 1.2 μM	10C
Full-length	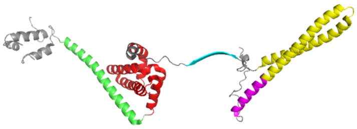	sBraf L597A	*Kd* = 0.23 μM	10D
Full-length	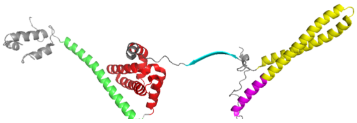	sBraf L597R	NB	10E
Full-length	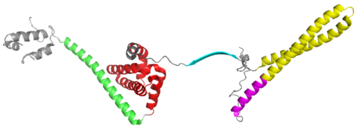	sBraf L597R-V600E	*K*d = 0.34 μM	11D

Supplementary Figure S1 Constructs used in this study.

Refer to Figure 2A for amino acid positions of the various structural elements of Cdc37. *K*d values and the figure that each experiment represents is indicated.
